# Application of a dye-based mitochondrion-thermometry to determine the receptor downstream of prostaglandin E_2_ involved in the regulation of hepatocyte metabolism

**DOI:** 10.1038/s41598-018-31356-y

**Published:** 2018-08-30

**Authors:** Lei Shen, Tao-Rong Xie, Run-Zhou Yang, Yan Chen, Jian-Sheng Kang

**Affiliations:** 1 Key Laboratory of Nutrition, Metabolism and Food Safety, Shanghai Institute of Nutrition and Health, Shanghai Institutes for Biological Sciences, University of Chinese Academy of Sciences, Chinese Academy of Sciences, Shanghai, 200031 China; 2 Institute of Neuroscience and State Key Laboratory of Neuroscience, Shanghai Institutes for Biological Sciences, Chinese Academy of Sciences, Shanghai, 200031 China; 3 The First Affiliated Hospital of Zhengzhou University, Zhengzhou, 450052 China

## Abstract

Temperature distributions inside a living cell reflect the thermodynamics and functions of cellular components. We used a newly-developed method of mitochondrial thermometry based on Rhodamine B methyl ester, which equilibrates as a thermosensitive mixture of nonfluorescent and fluorescent resonance forms. Prostaglandin E_2_ (PGE_2_) is released from hepatic non-parenchymal Kupffer cells and acts as an inflammatory factor to impact various functions of hepatocytes. The activity of PGE_2_ on energy mechanism of hepatocytes has not been fully elucidated and in particular, which PGE_2_ receptor mediates the functions has been elusive. We identified EP4 as the major receptor of PGE_2_ via our mitochondrion-thermometry approach and then substantiated this receptor’s role in hepatic metabolism. We discovered that PGE_2_ is able to decrease intracellular temperature of hepatocytes, via increasing some lipogenic genes’ expressions, hampering lipolysis and mitochondrial β-oxidation, reducing intracellular ATP level and elevating cAMP level through EP4 receptor. The redox status of hepatocytes represented by FAD vs FAD + NADH ratio is influenced by PGE_2_ in an EP4 receptor-dependent manner. Collectively, these data demonstrate that PGE_2_ regulates metabolism of hepatocytes mainly through EP4 receptor.

## Introduction

Current understanding of metabolic rate, one of physiological processes and mechanisms of evolutionary adaption to temperature change, indicates that temperature and metabolic rate are indirectly influenced^1^. Temperature is a fundamental physical quality that governs every biological reaction within living cells^2–5^. The biological reactions responsible for cellular functions occur either exothermically or endothermically at particular locations within a cell, such as organelles. Hence, temperature distributions inside a living cell reflect the thermodynamics and functions of organelles^6^. Recently, the quantification of intracellular temperature has been put forwarded as it correlates with biochemical reaction rates. A couple of methods have been established to detect intracellular temperature including our ratiometric method^7–13^. Cellular pathogenesis of disease like cancer is characterized by extraordinary heat production^14^. Thus, better understanding of intracellular temperature will lead us to establish novel diagnosis and therapies for some diseases to some extent. Fluorescence intensity-based measurements are influenced by fluid optical properties, dye concentration and other experimental or systematic factors. A ratiometric approach avoids most interferences^15,16^. Though influenced by viscosity, the ratios are independent of the dye concentration or other factors^17–20^. One of the most important merits of the method is that it is non-invasive and can monitor how intracellular temperature changes with external stimulus in a time-dependent manner.

In the liver, PGE_2_ is mainly produced by Kupffer cells and acts as an important lipid mediator of inflammation to hepatocytes^21^. A number of studies have indicated that PGE_2_ is able to modulate the metabolism of hepatocytes in many ways. PGE_2_ stimulates lipid accumulation in hepatocytes by reducing hepatic lipolysis, mitochondrial β-oxidation and VLDL synthesis^22^. It is interpreted that PGE_2_ is mediated by PGC1α as that the over-expression of PGC1α could attenuate PGE_2_-induced lipid accumulation in hepatocytes^22^. Previous reports demonstrated that PGE_2_ is able to reduce VLDL secretion in primary hepatocytes^23,24^. In addition, PGE_2_ was found to be involved in alcohol-induced hepatic steatosis due to stimulation of PGE_2_ production in Kupffer cells^25^.

Although PGE_2_ was found to affect cellular metabolism in the liver cell and some studies applied animal models via genetic manipulations to study the physiological functions of PGE_2_ receptors^26–29^, it is still unknown about the nature of the major receptor that mediates the effect of PGE_2_ within hepatocytes in a natural state. PGE_2_ must exert its biological effect through one of its four receptors namely EP1, EP2, EP3, and EP4, in a physiological condition. In addition, how PGE_2_ affects the basic features of cellular metabolism of hepatocytes such as the intracellular temperature, ATP level and redox status is unclear.

First, mitochondrion is targeted for representing intracellular temperature because it plays a key role in maintaining thermal homeostasis in the cell^30^. We applied a mitochondrial thermometry (mito-thermometry) based on the thermo-sensitive characteristics of Rhodamine B methyl ester (RhB-Me)^13^. With this approach, we could demonstrate how PGE_2_ affects hepatocyte’s intracellular activities and determine which receptor regulates the intracellular events. Secondly, biological effects of PGE_2_ on energy metabolism in hepatocytes were substantiated to demonstrate the link between intracellular thermodynamics and biological processes. Therefore, our work broadens our understanding about the regulatory activities of PGE_2_ on the metabolism of hepatocytes.

## Results

### PGE_2_ decreases intracellular temperature of hepatocytes via EP4 receptor

Mitochondrion is the main intracellular site for thermogenesis. We applied the mito-thermometry based on RhB-Me which equilibrate as a thermosensitive mixture of nonfluorescent and fluorescent resonance forms as recently reported^13^. Although fluorescence dyes might bleach after long-time imaging, we found that the fluorescence remained fairly stable (Supplemental Fig. 1). Similarly, the solvent had no effect on the intracellular temperature in primary hepatocytes (Supplemental Fig. 2). We measured the thermal response in mitochondria of hepatic cells to confirm the validity and efficacy of the mito-thermometry method. Carbonyl cyanide 3-chlorophenylhydrazone (CCCP), an uncoupling reagent of oxidative phosphorylation, is known to generate thermogenesis in mitochondria^31–33^. As expected, CCCP caused a slow increase of intracellular temperature and then reached a plateau at a later stage (Supplemental Fig. 3A). Thermoneutral Rh800 and thermosensitive RhB-Me were both accumulated in the mitochondria (Supplemental Fig. 3B). The fluorescent intensity of both RhB-Me and Rh800 attenuated upon CCCP treatment, but RhB-Me’s fluorescence intensity is dropping much more dramatically than Rh800′s. This led to the ratio of Rh800 to RhB-Me increased, indicating that CCCP dissipated electrochemical potential stored in mitochondrial proton gradient, depolarized mitochondrial membrane potential, converted the energy to heat, and increased mitochondrial temperature as expected (Supplemental Fig. 3A). To visualize intracellular temperature, we made a pseudo ratio image to demonstrate the changes of intracellular temperature before and after treatment (Supplemental Fig. 3B, right column). Now we could state that this method also works well in hepatic cells.

**Figure 1:**
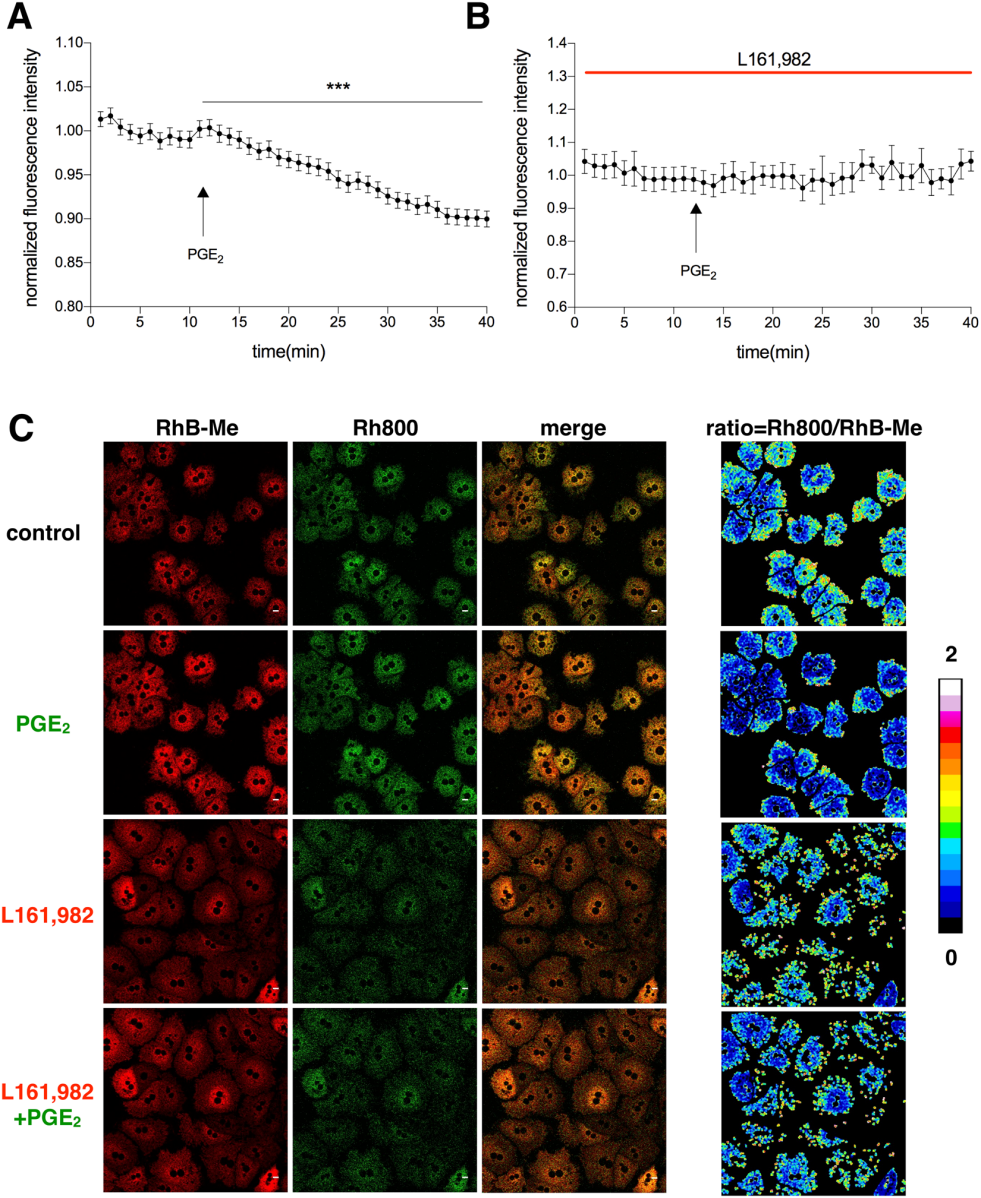
PGE_2_ reduces intracellular temperature of primary hepatocytes via EP4 receptor. (**A**) Primary hepatocytes were stained with both RhB-Me (20 nM) and Rh800 (20 nM) in tyrode solution for 90 minutes using 559 nm and 633 nm excitation wavelength respectively. The ratio was calculated by dividing the fluorescence of Rh800 to RhB-Me. PGE_2_ (10 nM) was applied to the tyrode solution at the indicated time. (**B**) EP4 antagonist L161, 982 (1 μM) was added to the tyrode solution 2 hours before confocal imaging. PGE_2_ (10 nM) was injected to the buffer at the indicated time point. (**C**) The fluorescence intensity of RhB-Me and Rh800 before and after PGE_2_ treatment (10 nM), without or with pre-incubation with L161, 982 (1 μM). The pseudo-color images of the intensity ratio of Rh800 to RhB-Me are shown in the right panels. Scale bars, 10 μm. The data are shown as mean ± SEM with *** for p < 0.001. The same experiments were repeated at least three times with similar results.

To determine how PGE_2_ affects the intracellular temperature of hepatocytes, we applied this fluorescent ratiometric strategy to investigate how PGE_2_ affects the intracellular temperature of hepatocytes. Consequently, we found that PGE_2_ caused ~10% drop of the fluorescence ratio in primary hepatocytes (Fig. 1A). We next analyzed which PGE_2_ receptor mediates the effect of PGE_2_ on the thermal response of hepatocytes. The fluorescent intensity of RhB-Me enhanced dramatically upon PGE_2_ treatment while the intensity of Rh800 remained almost constant, indicating that mitochondrial membrane potential was not affected while thermal effect took shape (Fig. 1C). Pre-incubation of the cells with L161, 982, an EP4 antagonist, was able to completely abrogate PGE_2_-mediated fluorescent ratio drop in hepatocytes (Fig. 1B). However, antagonists of EP1, EP2 and EP3 could not block the effect of PGE_2_-mediated decrease of the ratio (Supplementary Fig. 4A,B). The fluorescent intensities of RhB-Me increased and the intensities of Rh800 remained constant (Supplemental Fig. 4C,D). Hence, these observations indicate that PGE_2_ is able to decrease hepatic intracellular temperature via EP4 receptor.

### PGE_2_ regulates lipid metabolism through EP4 receptor in hepatocytes

**Figure 2:**
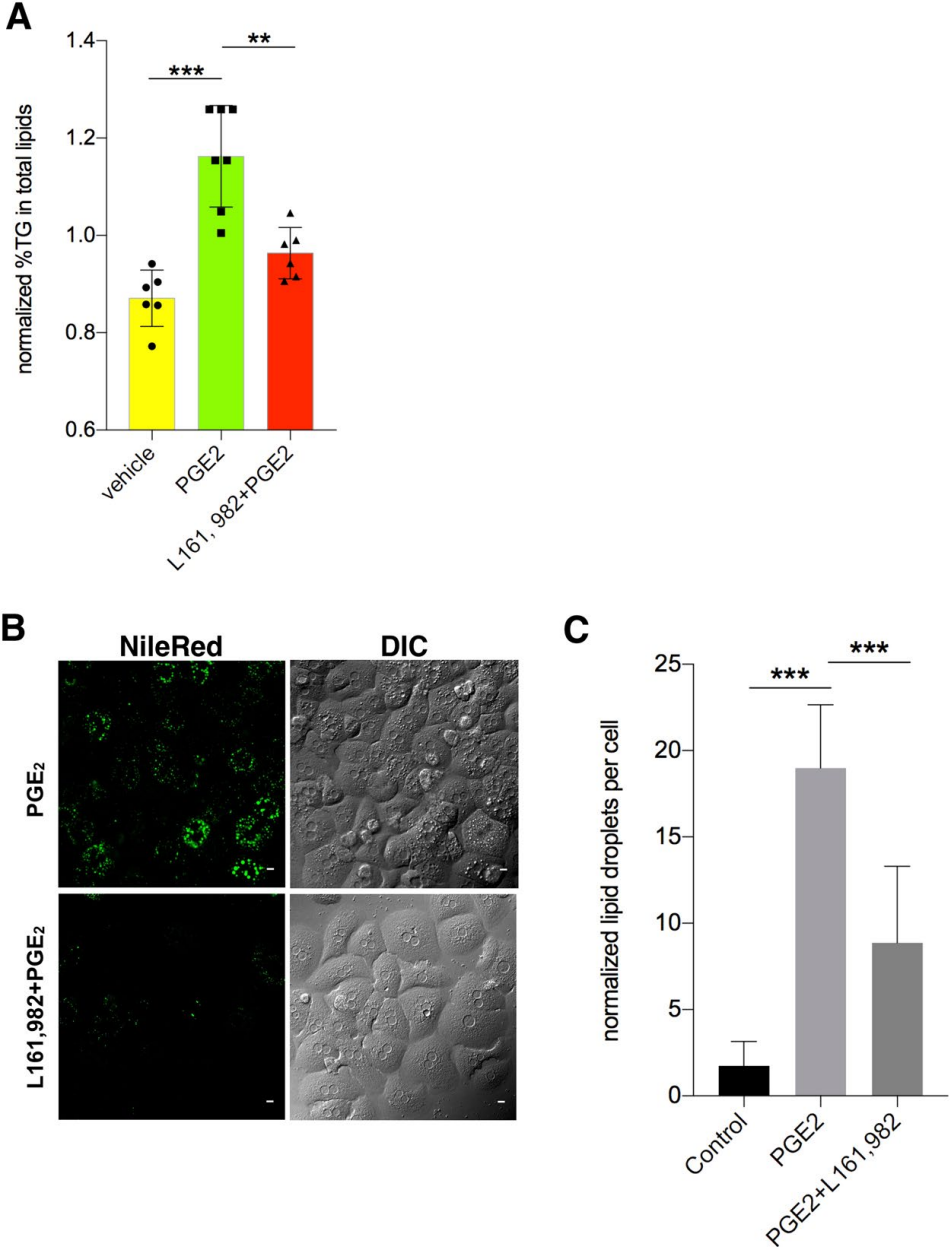
PGE_2_ enhanced lipogenic metabolism in hepatocytes. (**A**) TG level treated with PGE_2_ (10 nM) without and with L161, 982 (1 μM). (**B**) Cells treated with PGE2 and/or L161, 982 for 12 hours and then changed to tyrode solution. Cells were stained with NileRed (1 μM) for 30 minutes in tyrode solution in the incubator. Scale bars, 10 μm. (**C**) Quantification of the fluorescence intensity of NileRed. The data are shown as mean ± SEM with * for p < 0.05, ** for p < 0.01, *** for p < 0.001. The same experiments were repeated at least three times with similar results.

**Figure 3:**
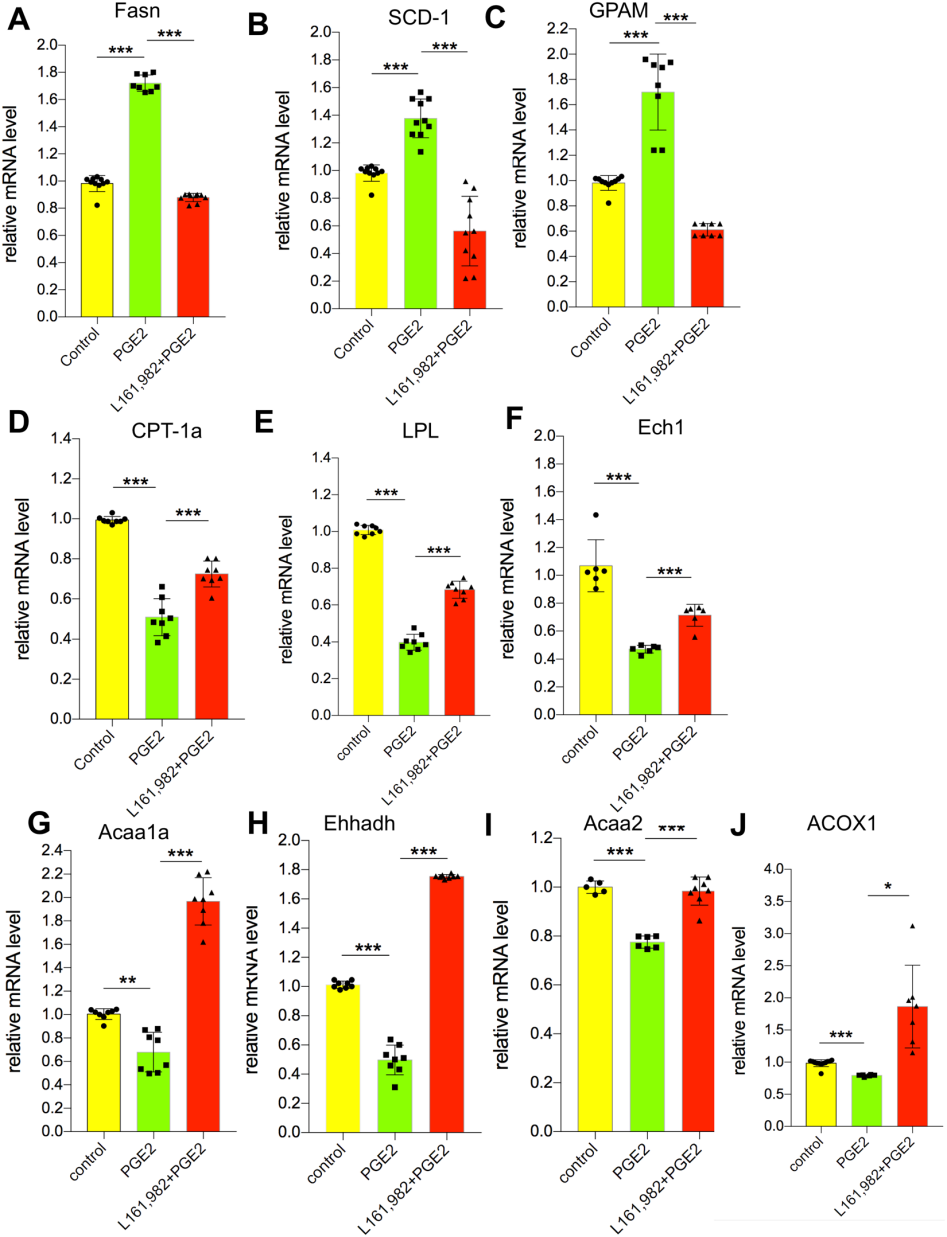
PGE_2_ regulates lipid metabolism through EP4 receptor in hepatocytes. (**A**) Relative mRNA level of Fasn. (**B**) Relative mRNA level of SCD-1. (**C**) Relative mRNA level of GPAM. (**D**) Relative mRNA level of CPT-1a. (**E**) Relative mRNA level of LPL. (**F**) Relative mRNA level of Ech1. (**G**) Relative mRNA level of Acaa1a. (**H**) Relative mRNA level of Ehhadh. (**I**) Relative mRNA level of Acaa2. (**J**) Relative mRNA level of Acox1. The data are shown as mean ± SEM with * for p < 0.05, ** for p < 0.01. The data are shown as mean ± SEM with *** for p < 0.001. The same experiments were repeated at least three time with similar results.

Intracellular temperature or overall body temperature is somehow related to metabolism. The combination of the mass scaling and temperature components led Gillooly *et al*. to propose a simple equation describing the variation of metabolic rate (Q) of all organisms: Q = b_0_M^3/4^e^−E/kT^, where M is body mass, T is absolute temperature, k is Boltzmann’s constant, E is the averaged activation energy of metabolism and b_0_ is a scaling constant independent of M and T^34^. A conceptual model of the relationship between resting metabolic rate and temperature in ectotherms was proposed^1^. Furthermore, previous studies have reported that PGE_2_ is able to induce lipid accumulation in primary hepatocytes^22^. Thus, to further consolidate our observation in PGE_2_’s regulation in hepatic intracellular temperature, we next investigated whether the effect of PGE_2_ on lipid metabolism in hepatocytes was also mediated by EP4. Consistently, we found that PGE_2_ significantly elevates hepatic triglyceride (TG) contents (Fig. 2A). Blockade of EP4 completely abolished the effect of PGE_2_ on TG production (Fig. 2A). We also found that more lipid droplets were accumulated in hepatocytes after PGE_2_’s treatment overnight (Fig. 2B). Likewise, blockade of EP4 attenuated lipid droplets accumulation (Fig. 2B). EP4 inhibitor could lower almost 1 fold of lipid droplets (Fig. 2C). Next, we investigated whether PGE_2_ elevated lipid synthesis and down-regulated lipolysis processes, since PGE_2_ elevated TG contents in hepatocytes. PGE_2_ was able to upregulate lipogenic genes including fatty acid synthase (Fas), stearoyl-CoA desaturase-1 (SCD-1) and mitochondrial glycerol-3-phosphate acyltransferase 1 (GPAM) (Fig. 3A–C). On the other hand, PGE_2_ could reduce the expression of genes involved in fatty acid oxidation and lipolysis such as carnitine palmitoyltransferase 1A (CPT-1a), lipoprotein lipase (LPL), delta(3,5)-delta(2,4)-dienoyl-CoA isomerase (Ech1), acetyl-coenzyme A acyltransferase 1A (Acaa1a), enoyl-CoA hydratase and 3-hydroxyacyl CoA dehydrogenase (Ehhadh), acetyl-coenzyme A acyltransferase 2 (Acaa2), and peroxisomal acyl-coenzyme A oxidase 1 (Acox1) (Fig. 3D–J). Together, these results suggest that PGE_2_ induces lipid accumulation in hepatocytes by enhancing lipid synthesis and impeding fatty acid oxidation. Consistent with our observation of the intracellular temperature of hepatocytes, we found that the PGE_2_-mediated regulations of these lipogenic genes or lipolysis genes either attenuated or enhanced by EP4 inhibitor (Fig. 3), and thus demonstrated that PGE_2_ regulates lipid metabolism of hepatocytes by EP4.

### Cytosolic ATP is reduced by PGE2 in an EP4-dependent manner

**Figure 4:**
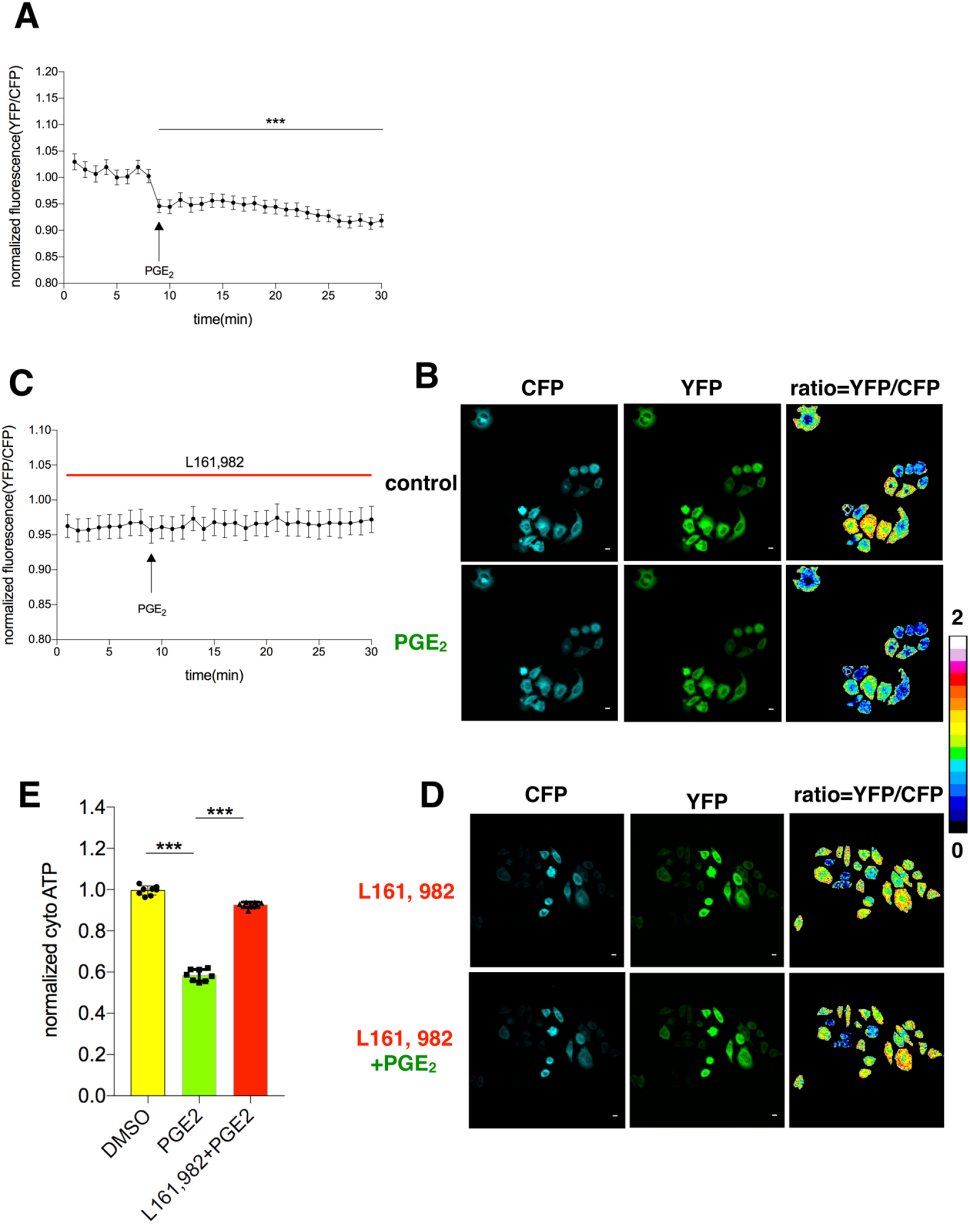
Cytosolic ATP is reduced by PGE_2_ in an EP4-dependent manner. (**A**) HepG_2_ cells electroporated with AT1.03 plasmid were changed from culture medium to tyrode solution and remained in there for 40 minutes before confocal imaging. PGE_2_ (10 μM) was applied at the indicated time. Fluorescence intensity of YFP and CFP were calculated to evaluate the intracellular ATP level. (**B**) Confocal images of the indicated channels before and after PGE_2_ treatment as in A. (**C**) The cells were incubated with L161, 982 (1 μM) and PGE_2_ (10 μM) as indicated. Fluorescence intensity of YFP and CFP was calculated. (**D**) Confocal images of the indicated channels before and after PGE_2_ treatment as in C. (**E**) ATP ELISA kit was applied to measure cytosolic ATP in primary hepatocyte according to manufacturer’s instructions. The cells were treated with PGE_2_ (10 nM) and L161, 982 (1 μM) for 12 hours as indicated before the measurement. The data are shown as mean ± SEM with * for p < 0.05, ** for p < 0.01, *** for p < 0.001. The same experiments were repeated at least three times with similar results.

As we found that the cellular temperature was affected by PGE_2_ in hepatocytes, we next investigated whether the intracellular ATP level was influenced by PGE_2_ since ATP is the major energy source of the cells to impact temperature and metabolism. We transfected HepG_2_ cells with the plasmid AT1.03 which is a genetically-coded fluorescence resonance energy transfer (FRET)-based ATP indicator^35^. Consistent with our observation that intracellular temperature was decreased by PGE_2_, an ATP indicator, the YFP/CFP ratio was decreased by about 10% upon PGE_2_ stimulation (Fig. 4A,B). In addition, blocking EP4 abrogated the effect of PGE_2_ (Fig. 4C,D). To further consolidate the results, we applied a biochemistry method. PGE_2_ caused about 40% drop of intracellular ATP level and EP4 antagonist abrogated such effect as well (Fig. 4E). The sensitivity of the plasmid may not be better than ELISA kit, which detected more dramatic ATP drop. Collectively, these data indicate that PGE_2_ is able to reduce intracellular ATP level via EP4 receptor.

### PGE_2_ affects hepatic mitochondrial r**e**dox status through EP4 receptor

**Figure 5:**
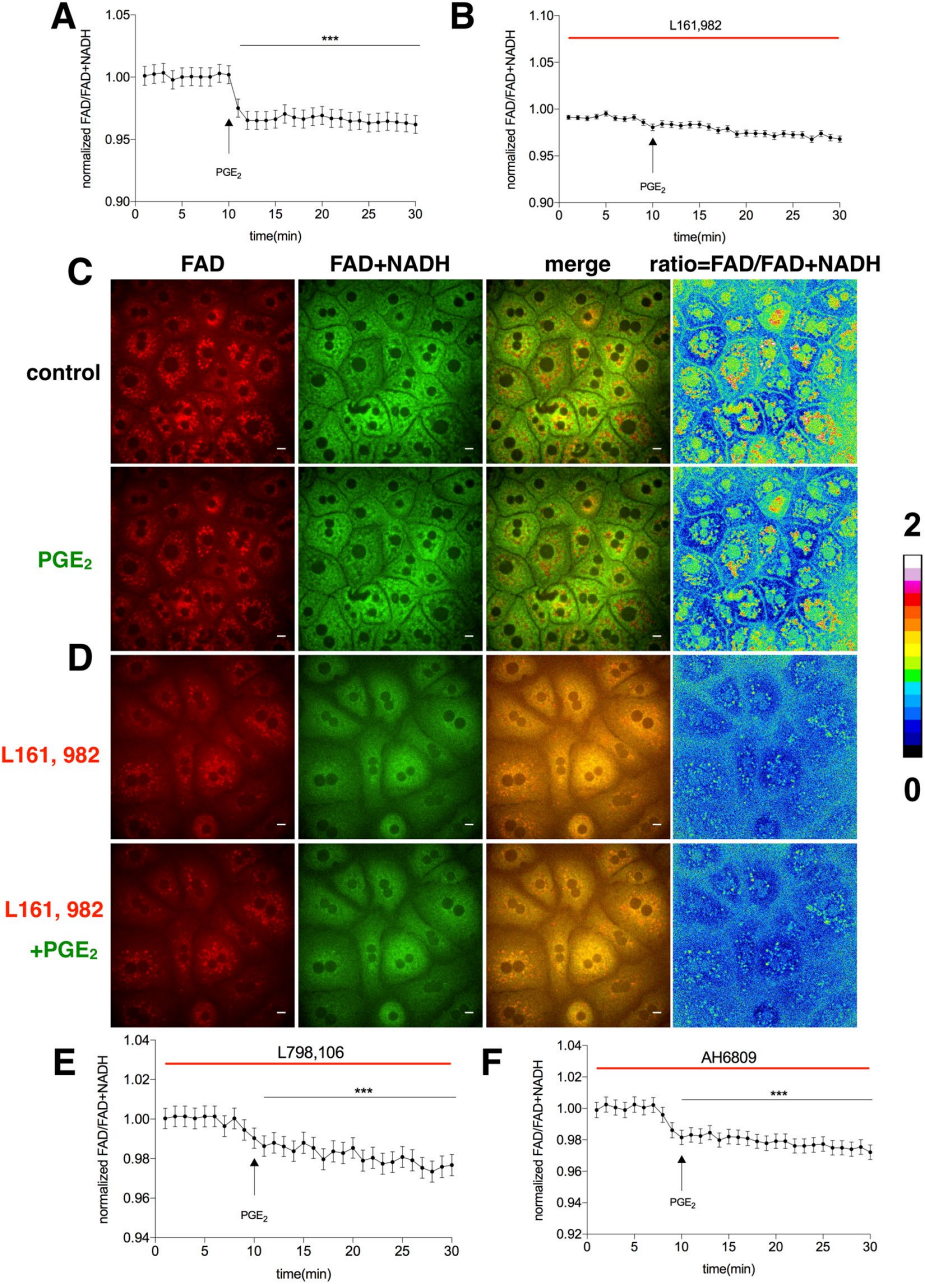
PGE_2_ affects hepatic mitochondrial redox status through EP4 receptor. (**A**) Primary hepatocytes were changed from culture medium to tyrode solution for 40 minutes before imaging. PGE_2_ (20 μM) was injected into the solution as indicated. The fluorescence intensity of FAD was divided by that of FAD + NADH to represent redox status of the cells. (**B**) Primary hepatocytes were treated with L161, 982 (1 μM) and PGE_2_ (20 μM) as indicated, followed by measurement of fluorescence ratio of FAD vs FAD + NADH. (**C**,**D**) The fluorescence images for A and B respectively. Scale bars, 10 μm. The data are shown as mean ± SEM with *** for p < 0.001. The same experiments were repeated at least three times with similar results.

As we found that PGE_2_ could lower intracellular temperature, improve lipid synthesis and reduce cytosolic ATP, we next explored whether the two key coenzymes, FAD and NADH were influenced by PGE_2_. NADH and FAD are two coenzymes involved in key metabolic pathways such as glycolysis, the Krebs cycle, and oxidative phosphorylation. These coenzymes are directly involved in ATP production through oxidative phosphorylation and exist in either oxidized (NAD+/FAD) or reduced (NADH/FADH_2_) forms. However, only NADH and FAD can yield significant fluorescence^36,37^. During glycolysis, two NAD + molecules gain electrons and are reduced to two NADH molecules, as one glucose molecule is broken down to two pyruvate molecules whereas an FAD molecule is reduced. The process of glycolysis produces a net gain of two ATP molecules^38^. 2-Deoxy-D-glucose (2DG) is a modified glucose molecule with 2-hydroxyl group being replaced by hydrogen, so that it cannot undergo further glycolysis. As such, it acts to competitively inhibit the production of glucose-6-phosphate from glucose. To validate our system, 2DG was applied to the primary hepatocytes and exhibited a sharp increase in the ratio of FAD/FAD + NADH as expected (Supplemental Fig. 5A,C). Primary hepatocytes were starved for 1 hour and were then fed with glucose. The FAD/FAD + NADH ratio was reduced by resupplied glucose (Supplemental Fig. 5B,D). We next used this system to analyze the effect of PGE_2_ on hepatocytes. We found that PGE_2_ reduced the FAD/FAD + NADH ratio (Fig. 5A,C), indicating that PGE_2_ might enhance glycolysis, as there was a net increase in NADH level. Blocking EP4 could abrogate the inhibitory effect of PGE_2_ on the FAD/FAD + NADH ratio (Fig. 5B,D). Furthermore, blocking EP1, EP2 and EP3 had no effect on PGE_2_-mediated changes in the FAD/FAD + NADH ratio (Supplemental Fig. 6). These data, therefore, indicate that EP4 mediates the effect of PGE_2_ on electron transport chain of mitochondria in hepatocytes, consistent with the above results.

### PGE_2_ regulates intracellular cAMP level via EP4 receptor

**Figure 6:**
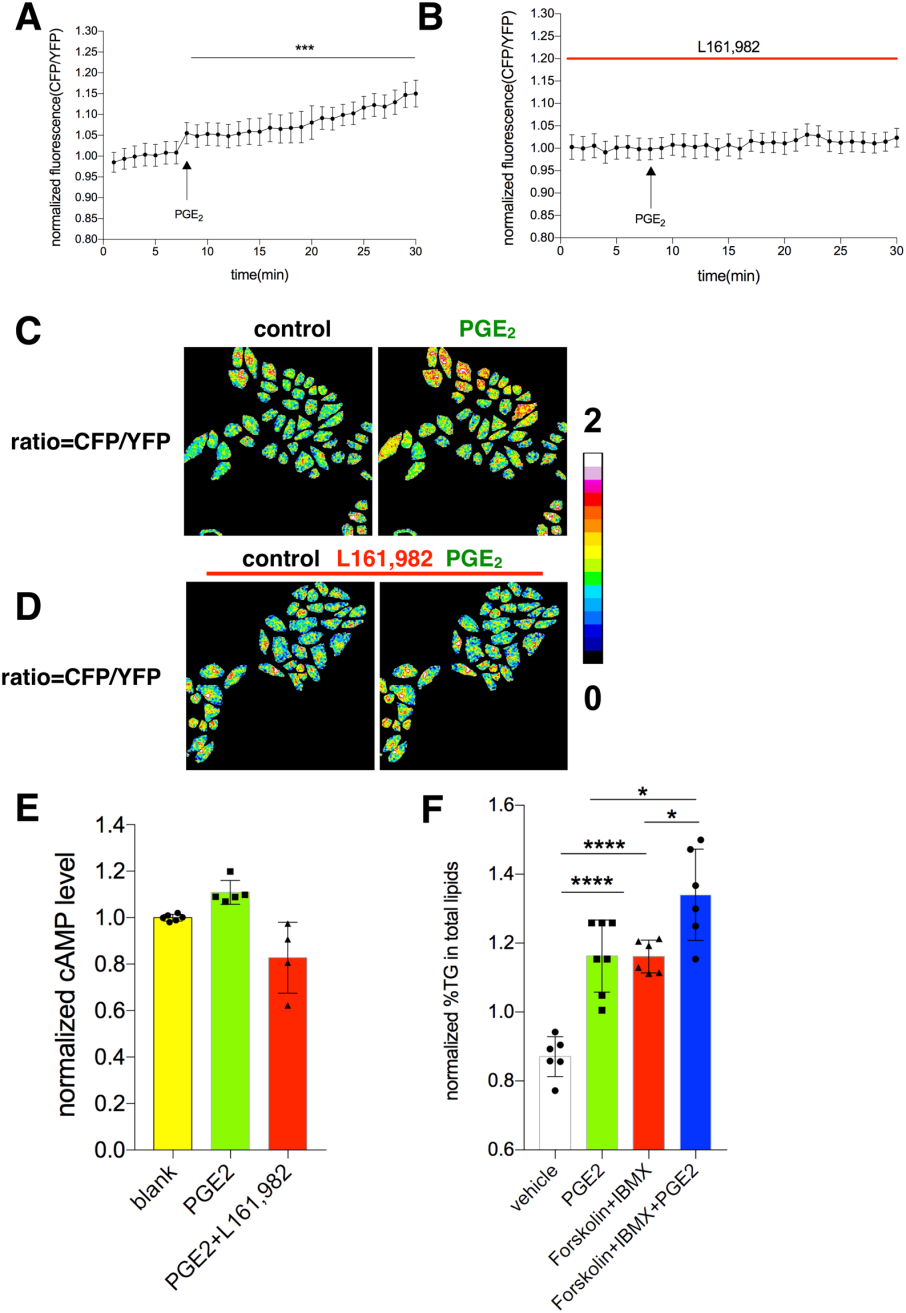
PGE_2_ regulates intracellular cAMP level via EP4 receptor. (**A**) HepG_2_ cells electroporated with H74 plasmid were changed from culture medium to remain in tyrode solution for 40 minutes before confocal imaging. The cells were treated with PGE_2_ (20 μM) as indicated. The fluorescence ratio of CFP vs YFP was calculated. (**B**) The cells were treated with L161, 982 (1 μM) before PGE_2_ treatment, followed by measurement of CFY/YFP ratio. (**C**,**D**) Pseudo-color image of intensity ratio before and after PGE_2_ (20 μM) without and with L161, 982 (1 μM). (**E**) Primary hepatocytes were treated with or without PGE_2_ (10 nM) and L161, 982 (1 μM) as indicated for 12 hours after the cells reached adherence. The cells were harvested for cAMP measurement following manufacturer’s instruction. The same experiments were repeated at least three times with similar results. (**F**) Triglyceride concentration of HepG2 cells with or without the treatment as indicated. The data are shown as mean ± SEM with * for p < 0.05, ** for p < 0.01, *** for p < 0.001 and **** for p < 0.0001.

EP4 receptor regulates hepatic intracellular temperature and we wondered what the underlying mechanism was. The classic downstream of Gs protein leads to cAMP increase. Upon activation by PGE_2_, EP4 couples with Gs and leads to production of cAMP^39^. We transfected HepG_2_ cells with a plasmid that acts as a cAMP sensor and used it to measure intracellular changes of cAMP^40^. As expected, PGE_2_ was able to raise intracellular level of cAMP for about 15% from the baseline (Fig. 6A). Blockade of EP4 abrogated the elevation of cAMP caused by PGE_2_ treatment (Fig. 6B). The pseudo ratio images also clearly illustrated the changes before and after PGE_2_’s treatment (Fig. 6C,D). In addition, we used a biochemistry method to measure intracellular cAMP level and confirmed that PGE_2_ was indeed able to increase cAMP level while inhibition of EP4 abrogated the effect (Fig. 6E). Enhanced cAMP level triggered more TG contents in hepatocytes either by forskolin or PGE2 treatments (Fig. 6F), which emphasized the PGE_2_-EP4-cAMP axis in regulating hepatic metabolism.

## Discussion

We applied the very sensitive method of dye-based mito-thermometry to determine PGE_2_’s receptor in regulating hepatocyte’s metabolism and revealed that EP4 is the major receptor mediated the regulatory roles of PGE_2_ on metabolism. Recently, a team of researchers with members from France, Korea and Germany has found that temperatures inside human mitochondria can be as high as 50 °C^41^. Their finding can explain to some extent the phenomenon that PGE_2_ decreases hepatocytes’ temperature (Fig. 1A). The high temperature of cellular power house, mitochondrion, reflects its function as factory that can produce heat. PGE_2_ downregulates biological activity and causes a decreased mitochondrial temperature, which is reasonable though provocative.

As for lipid metabolism, we found that PGE_2_ could enhance lipid synthesis and attenuate lipolysis process (Figs 2 and 3). This might help to understand that temperature and metabolic rate are somehow influenced. Prostaglandins may affect hepatocyte’s metabolism directly. Nevertheless, there are paradox reports about the impact of prostaglandins on hepatic lipid metabolism. Although some studies state that prostaglandins may favor fat accumulation in hepatocytes and thus lead to the development of hepatic steatosis^22^, others provide evidences that PGE_2_ might suppress de novo lipogenesis or that PGE_2_ does not affect lipogenesis but attenuate triglyceride incorporation into VLDL^23,24^. Our study indicates that PGE_2_ is able to induce lipid accumulation in hepatocytes by elevating lipid synthesis while impeding fatty acid oxidation. Furthermore, we found that the regulatory effect of PGE_2_ on lipid metabolism is mediated by EP4 receptor, which is consistent with the phenomenon that PGE_2_ lowered hepatocytes intracellular temperature through EP4 receptor.

In agreement with the notion that ATP is the major form of energy metabolism, PGE_2_ was able to reduce intracellular ATP level via EP4 receptor. With the genetically-encoded ATP sensor, we witnessed the lowering down ATP concentration after PGE_2_ treatment (Fig. 4A). EP4 could reverse the effect while other receptors couldn’t do so. Consistently, we found that the effect of PGE_2_ on the FAD vs NADH + FAD ratio in hepatocytes was also mediated by EP4 receptor. Using endogenous contrast to monitor FAD vs FAD + NADH to assess metabolic state is non-invasive and allows researchers to characterize metabolism without interfering the sample. A decrease in redox ratio (increasing NADH or/and decreasing FAD) could be explained by NADH elevation by inhibiting oxidative phosphorylation^38^. Consequently, the reductions of ATP level and redox ratio both suggest that PGE_2_ inhibits oxidative phosphorylation through EP4 receptor. Previous research showed that elevated NADH fluorescence and lowered redox ratio are characteristics of both aggressive tumors and differentiating stem cells synthesizing lipid droplets^38^. These results may explain why PGE_2_ induced lipid droplets in hepatocytes.

 While EP1 and EP2 have roles in pain/allergy or ovulation/fertilization respectively, EP3 was reported to regulate obesity and adipocyte metabolism^27–29^. EP4 can couple with Gs resulting in cAMP generation^39^. Consistently, we found that PGE_2_ could raise intracellular cAMP level through EP4 receptor in hepatocytes. Moreover, forskolin and IBMX can further enhance PGE_2_ to induce more hepatic TG contents. It is known that enhanced cAMP level could induce lipolysis in adipocytes, but signal is reversed in hepatocytes. There is a paradox in literatures about EP4 signaling, i.e., whether it is coupled to Gs or Gi at what circumstances^39^. In adipocytes, the increase of cAMP would lead to lipolysis via cAMP-PKA-HSL (hormone sensitive lipase). However, the increase of cAMP in hepatocytes increases TG level (Fig. 6F). Therefore, the PGE_2_-EP4-cAMP axis is able to stimulate lipid synthesis instead of lipolysis in hepatocytes. Enzymes involved in lipid metabolism of liver might also have their roles, like perilipin 1 and Acetyl-CoA carboxylase (ACC). Cross-interactions between liver, adipose tissue and muscle might also contribute to lipid accumulation in the liver. The axis we discovered is part of the liver’s lipid metabolism. Further delineating the regulatory role of PGE_2_-EP4-cAMP axis in hepatocytes might be helpful using liver-specific EP4 knockout mouse model.

Collectively, these findings demonstrated that PGE_2_ regulates various aspects of metabolism of hepatocytes through EP4 receptor. Our studies at the cellular level would pave the way for future characterization of PGE_2_ in the liver. In particular, due to the unique role of PGE_2_ in energy metabolism, it may create new strategies for the treatment of hepatic diseases in the future.

## Methods

### Materials

The materials were from following sources: Rhodamine B, Rhodamine 800, collagenase type II, forskolin, and D-glucose from Sigma-Aldrich (MO, USA); William’s E medium, fetal bovine serum and Hank’s Balanced Salt Solution from Thermo Fisher Scientific Co. Ltd. (Waltham, MA, USA); matrigel was from BD Biosciences Company (New York, USA); coverslips were from Glaswarenfabrik Karl Hecht GmbH & Co KG (Rhön, Germany); PGE_2_, L161, 982, L798, 106, AH6809 and 3,7-Dihydro-1-methyl-3-(2-methylpropyl)-1H-purine-2,6-dione(IBMX) from Cayman (Michigan, USA); Percoll from GE Healthcare (Freiburg, Germany); CCCP from ABCAM (Cambridge, MA, USA); ATP assay kit from Beyotime (Shanghai, China); cAMP assay kit from R&D systems (Minnesota, USA); Bradford reagent for protein concentration analysis from BIO-RAD (Berkeley, California, USA). Rhodamine B-Me was synthesized as recently reported^13^.

### Animals

C57BL/6J mice (from Shanghai SLAC Laboratory Animal Co., Ltd, Shanghai, China) had free access to regular chow diet and plain tap water for 8 weeks at a temperature of 22 °C with a 12-hour light/dark cycle. Treatment of the animals followed the Chinese animal protection laws and all experiments were approved by the ethics committee of the Institutional Animal Care and Use Committee of Shanghai Institutes for Biological Sciences, Chinese Academy of Sciences and the principles of laboratory animal care.

### Preparation and culture of hepatocytes

Density gradient-purified hepatocytes were prepared with the use of collagenase as described previously^42^. Briefly, parenchymal and non-parenchymal cells were isolated by two-step collagenase perfusion and purified by using Percoll gradients. The hepatocytes were plated on coverslips within a 35-mm diameter tissue culture plates (1 × 10^6^ cells/plate) in William’s E medium with 10% (v/v) fetal bovine serum at 37 °C and 5% CO_2_ in humidified atmosphere.

### Electroporation of HepG_2_ cells

HepG_2_ cells were electroporated according to T820/830 Electroporation Protocol (ElectroSquarePoratorTM). Briefly, HepG_2_ cells were trypsinized at 90% confluence and then centrifuged for 5 minutes at 500 g. The cell pellet was resuspended in PBS buffer. 20 μg of plasmid (H74, AT1.03, gifts, stated in Ackowledgement) was added. A 400 μL drop volume containing cells and plasmid was then placed between the electrodes with 4 mm gap connected to the electroporator (ECM 830 Square Wave Electroporation System, Holliston, MA, USA). For each electroporation, LV Mode (99 msec/500 V), 150 V voltage, 70 ms pulse length, 1 pulse were used. After electroporation, the cells were placed in a well of a 10 mm dish with coverslips inside and incubated in DMEM with 10% (v/v) fetal bovine serum at 37 °C and 5% CO_2_ in humidified atmosphere.

### Time-lapse imaging for mitochondrion-based thermometry

Thermo-sensitive confocal imaging was operated according to Xie *et al*.’s work^13^. Briefly, all time-lapse imaging was performed using confocal microscope with a 40×/0.95 objective (Olympus). Cells were co-stained with RhB-Me and Rh800 (20 nM) in Tyrode’s solution (in mM: 10 Hepes, 10 glucose, 3 KCl, 145 NaCl, 1.2 CaCl_2_, 1.2 MgCl_2_, pH 7.4) for 1 h in the cell incubator at 37 °C and 5% CO_2_. The pseudo color of RhB-Me channel is set as red color (excited at 559 nm and collected at 575–620 nm), and Rh800 channel is set as green color (excited at 635 nm and collected at 655–755 nm). All images were collected at 512 × 512 pixels resolution (12 bit).

### Hepatic triglyceride measurement

Triglyceride in hepatocytes was measured according to the TG Determination Kit (ShenSuoYouFu, Shanghai, China)^43,44^. Briefly, cells were digested and then resuspended in 600 μL DMEM. 100 μL DMEM containing partial cells were left for protein concentration measurement. The left 500 μL cell suspension was homogenized in 1.5 mL of a mixture of CHCl_3_-CH_3_OH (2:1, v/v), followed by shaking at room temperature for 2 h. After addition of 0.5 mL of 0.1 M NaCl, the suspension was centrifuged at 3,700 rpm for 10 min, and the lower organic phase was transferred and air-dried in a chemical hood overnight. The residual liquid was resuspended in 400 μL of 1% Triton X-100 in absolute ethanol, and the concentrations of triglyceride was analyzed using the above mentioned commercial kits for measurements. PGE_2_ and other pharmaceutical agents were applied to the cultured cells 12 hours before final analyzation.

### Lipid droplets staining and imaging

Cells were stained with NileRed (1 μM) in Tyrode’s solution for 30 minutes in the cell incubator at 37 °C and 5% CO_2_. The pseudo color of NileRed channel is set as green color (excited at 488 nm and collected at 600 nm). All images were collected at 512 × 512 pixels resolution (12 bit).

### Real-time quantitative RT-PCR

Total hepatocyte RNA was isolated using TRIzol reagent (Invitrogen, Carlsbad, CA). After treatment with RNase-free DNase I (Roche Applied Science, Penzberg, Germany), first-strand cDNA was synthesized with M-MLV reverse transcriptase and random hexamer primers (Invitrogen). Real-time quantitative PCR was performed with the SYBR Green PCR system (Applied Biosystems, Foster City, CA), using actin as an internal control for normalization. Primers used for each target gene were summarized in Supplementary Table 1.

### Cytosolic ATP and cAMP time-lapse imaging

HepG_2_ cells were electroporated with plasmid AT1.03 and H74 for cytoplasmic ATP and cAMP visualization through ECM 830 Square Wave Electroporation System (BTX, MA, USA) respectively. After cells reach adherence after electroporation (24 hours after electroporation), cells were changed to tyrode solution and incubated at 37 °C, 5% CO_2_ for 40 minutes before imaging. Time lapse imaging were taken with Olympus FV1000 confocal system using a 40×, 0.95 n.a air immersion objective with a pinhole of 800. CFP channel is excited at 440 nm and collected at 480–495 nm whereas YFP excited at 440 nm and collected at 505–605 nm. Ratio metric value of YFP to CFP was calculated pixel by pixel to represent the relative cytosolic ATP quantity within the specimen. Ratio metric value of CFP to YFP was calculated pixel by pixel to represent the cAMP quantity within the specimen. The imaging parameters, data acquisition and analysis for cytosolic ATP and cAMP were the same as those in the thermo-sensitive imaging. The experiments were performed in triplicates and the results were presented as mean ± standard error.

### Redox ratio (FAD/FAD + NADH) measurement

Redox (FAD/FAD + NADH) imaging was performed using a customized fluorescence microscope with a 40/0.8 W objective (Olympus) for time-lapse imaging. The endogenous autofluorescence images were excited with Optoscan monochromator (Cairn Research Ltd., UK). FAD channel is excited by 430 nm light with 20 nm bandwidth and collected with 525–575 nm band pass filter whilst NADH + FAD channel sequentially excited by 340 nm light with 20 nm bandwidth and collected with 420 nm long pass filter. The fluorescence images were acquired with Evolve 512 EMCCD (Photometrics Ltd., UK). Time-lapse images of the cells were performed in 4 mL Tyrode’s solution at 33 °C. MATLAB (MathWorks Inc. USA) and ImageJ (NIH, USA) were applied to analyze images.

### Cytosolic ATP measurement via biochemistry method

ATP assay kit was from Beyotime and the assay was performed according to manufacturer’s instruction. After centrifugation to remove cell debris, the supernatant was added to the substrate solution. The experiments were performed in triplicates and the results were presented as mean ± standard error. Protein concentration was measured by using NanoDrop A280 (ThermoFisher, Waltham, MA, USA) to normalize final ATP quantification.

### Measurement of cAMP via biochemistry method

Levels of cAMP were measured by ELISA kit (R&D) by following its manual.

### Quantification of lipid droplets

Lipid droplets were quantified using image J (NIH, USA). Briefly, adjust and remove the backgrounds and read the fluorescence intensity of NileRed.

### Data acquisition and analysis for time-lapse imaging

Analyses were acquired according to Xie *et al*.^13^.

### Statistical analysis

All the experiments were performed at least three times, and p-values were calculated using Student’s t tests. All data points in figures represent mean ± S.E.M.

## Electronic supplementary material

Supplemental Materials
