# Supplementary material for: Application of a dye-based mitochondrion-thermometry to determine the receptor downstream of prostaglandin E2 involved in the regulation of hepatocyte metabolism
Source: Sci Rep. 2018 Aug 30;8:13065. doi: 10.1038/s41598-018-31356-y (PMC6117307; doi:10.1038/s41598-018-31356-y)
Supplement: Supplementary file 1 — Supplemental Materials [file 41598_2018_31356_MOESM1_ESM.pdf]

Supplemental Materialss

Application of a dye-based mitochondrion-thermometry to determine the receptor downstream of prostaglandin E_2 involved in the regulation of hepatocyte metabolism

Lei Shen, Tao-Rong Xie, Run-Zhou Yang, Yan Chen, and Jian-Sheng Kang

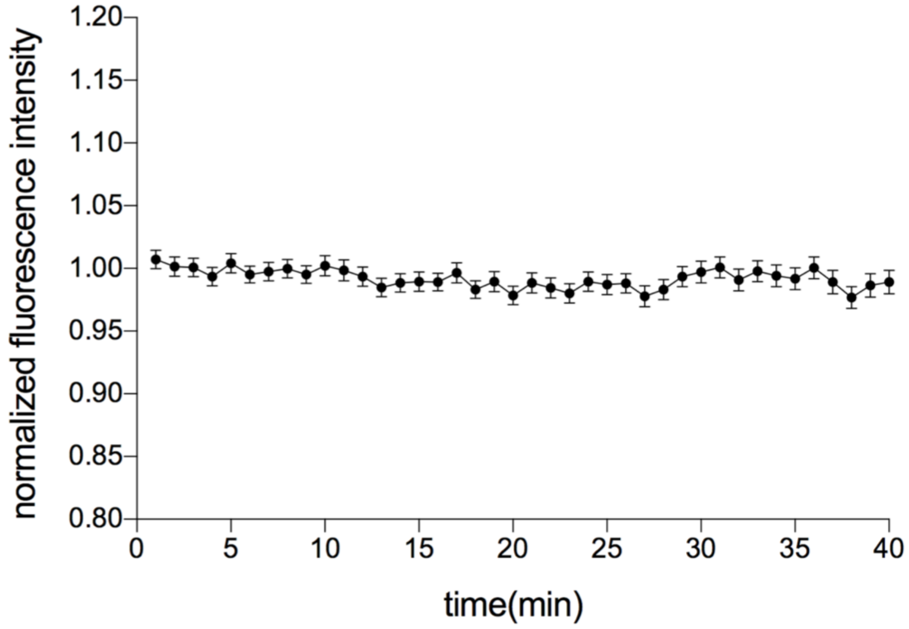

Supplemental Figure 1. Fluorescence signals of RhB-Me vs Rh800 are stable in long period

Primary hepatocytes were stained with both RhB-Me (20nM) and Rh800 (20nM) in tyrode solution for 90 minutes, 559nm and 633nm excitation wavelength respectively. No additional stimulation was injected to the solution. The data are shown as mean ± SEM.

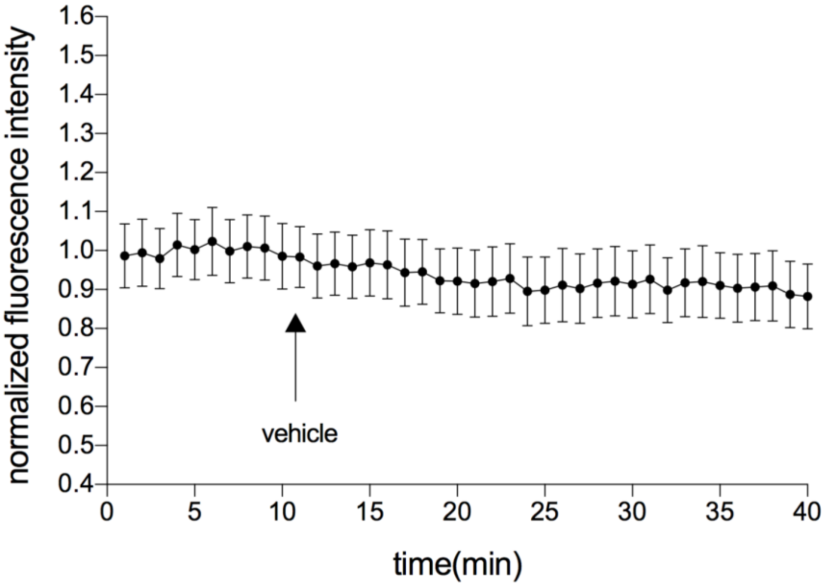

Supplemental Figure 2. Fluorescence signals of RhB-Me vs Rh800 are not altered by solvent

Solvent of PGE_2 was injected into tyrode solution at indicated time. The data are shown as mean ± SEM.

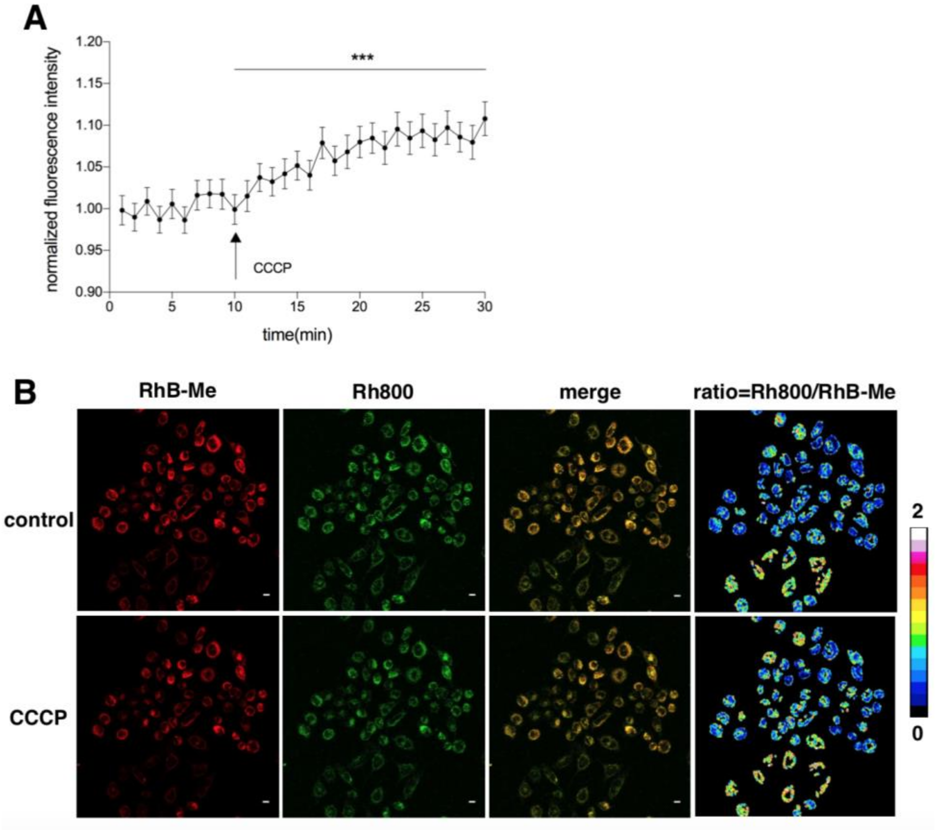

Supplemental Figure 3. The fluorescence intensity of RhB-Me and Rh800 before and after CCCP treatment

(**A**) $HepG_2$ cells were stained with both RhB-Me (20 nM) and Rh800 (20 nM) in tyrode solution for 90 minutes using 559nm and 633nm excitation wavelength respectively. The ratio was calculated by dividing the fluorescence of Rh800 to RhB-Me. CCCP (1 μM) was applied to the tyrode solution at the indicated time. The data are shown as mean ± SEM with *** for $p < 0.001$. (**B**) Primary hepatocytes were treated without or with CCCP (1 μM). Scale bars, 10 μm. The right column shows mitochondrial thermal map of hepatocyte represented by the pseudo-color image of intensity ratio of Rh800 to RhB-Me.

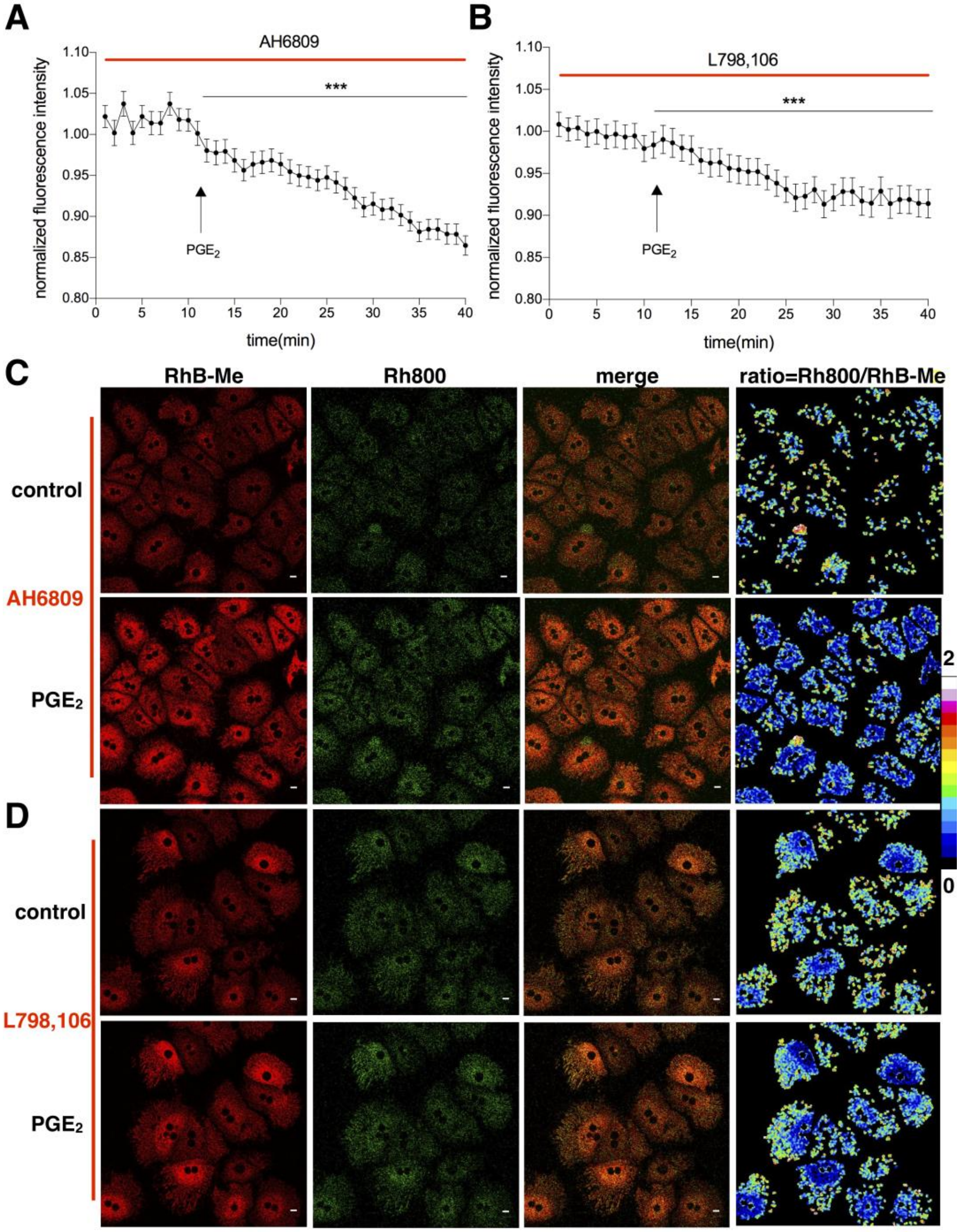

Supplemental Figure 4. EP1, EP2 and EP3 receptors are not involved in the reduction of hepatic intracellular temperature caused by PGE_2

(**A**) Primary hepatocytes were stained and analyzed as in Figure 1. EP1 and EP2 antagonist AH6809 (1 μM) was added to the tyrode solution 2 hours before confocal imaging. PGE_2 (10 nM) was injected to tyrode solution at the indicated time point. Error bars are represented as mean ± SEM. Significance was determined by Student's t test. (**B**) EP3

antagonist L798, 106 (1 μM) was added to the tyrode solution 2 hours before confocal imaging. PGE_2 (10 nM) was injected to tyrode solution at the indicated time point. (**C, D**) The fluorescence intensity of RhB-Me and Rh800 before and after PGE_2 treatment (10 nM), with or without pre-incubation with AH6809 (1 μM) or L798, 106 (1 μM). The pseudo-color images of the intensity ratio of Rh800 to RhB-Me are shown in the right panels. Scale bars, 10 μm. The data are shown as mean ± SEM with *** for $p < 0.001$.

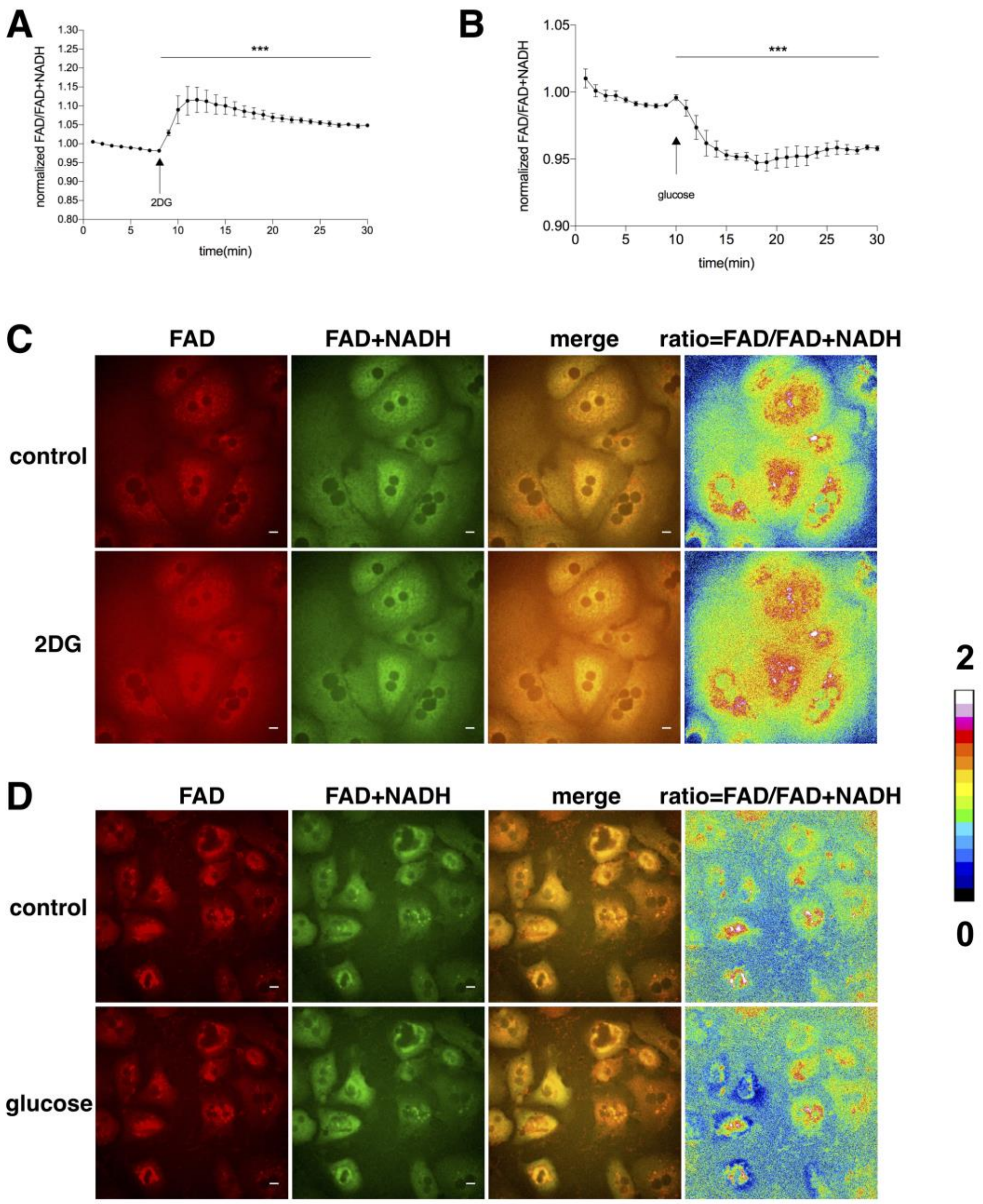

Supplemental Figure 5. Control experiments for redox status measurement in hepatocytes

(**A, B**) Primary hepatocytes were changed from culture medium to remain in tyrode solution for 40 minutes before imaging. 2DG (10 mM) or glucose (20 mM) was injected into the solution as indicated. Ratio was calculated through the fluorescence intensity of

FAD divided by that of FAD+NADH. The data are shown as mean ± SEM with *** for $p < 0.001$. (**C, D**) The fluorescence intensity of FAD and FAD+NADH before and after 2DG or glucose's injection into the solution. Scale bars, 10 μm. The pseudo-color images of the intensity ratio of FAD to FAD+NADH are shown in the right panels.

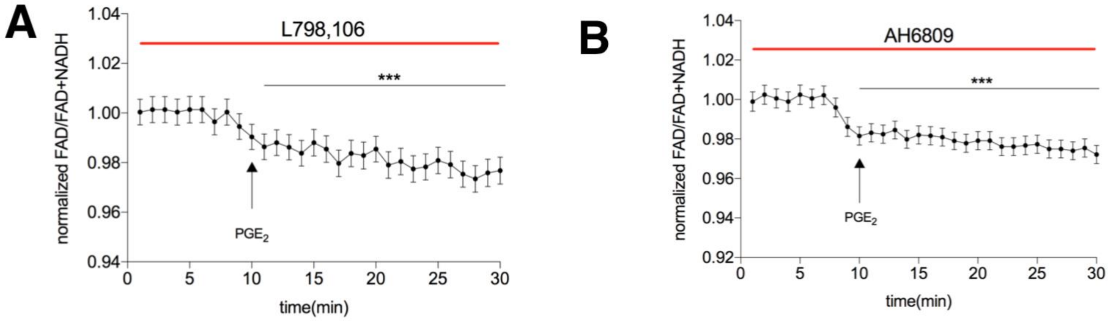

Supplemental Figure 6. EP1, EP2 and EP3 receptors aren't involved in hepatic mitochondrial redox status

(**A**) Primary hepatocytes were treated with L798, 106 (1 μM) and PGE_2 (20 μM) as indicated, followed by measurement of fluorescence ratio of FAD vs FAD+NADH.

(**B**) Primary hepatocytes were treated with AH6809 (1 μM) and PGE_2 (20 μM) as indicated, followed by measurement of fluorescence ratio of FAD vs FAD+NADH.

The data are shown as mean ± SEM with *** for $p < 0.001$. The same experiments were repeated at least three times with similar results.

Supplemental Table 1. Primer sequences used in RT-PCR

Gene	forward primer(5'-3')	reverse primer(5'-3')
FAS	CTATGGATTACCCAAGCGG	AGTGTTCGTTCCTCGG
CPT-1a	CTCCGCTCGCTCATTCC	ACACCCACCACCACGATAAG
ACOX1	GCGGTCCCTTGACCTTTTACC	CTGCAATCCCCGACGCT
Ehhadh	ATGATCCGCCTCTGCAATCC	GCTCCACAGATCACTATGGCT
SCD-1	GAAAGCCGAGAAGCTGGTGA	AACAGGAACTCAGAAGCCCAA
GPAM	AGCAAGTCCTGCGCTATCAT	CTCGTGTGGGTGATTGTGAC
LPL	GGGAGTTTGGCTCCAGAGTTT	TGTGTCTTCAGGGGTCCTTAG
Ech1	AAGATAAGGACGCCATGCTGAA	TCCAGGTGGCCATGTAGTCA
Acaa1a	ACGGTCAACAGACAGTGTTCA	GACCCATTTCTGATGCCACC
Acaa2	AGACCATGCAAGTGGACGAG	TTCCACCTCGACGCCTTAAC
β-actin	GATCATTGCTCCTCCTGAGC	ACTCCTGCTTGCTGATCCAC
